# Guilt and Shame of What Might Have Been in Optimistic Offender Drivers

**DOI:** 10.3389/fpsyg.2021.668138

**Published:** 2021-10-07

**Authors:** Carlos Hugo Criado del Valle

**Affiliations:** Faculty of Psychology, University of Salamanca, Salamanca, Spain

**Keywords:** offender drivers, optimism, counterfactual thinking, negative emotions, road safety

## Abstract

Habitual offender drivers are required to recover points lost on their driving license by attending reeducation courses, an experience that may, upon reflection of the incident in question, induce feelings of guilt or shame for the infractions they committed. A simulated driving task studied optimistic offender drivers to analyze the extent to which the controllability of the situational context influenced their use of internal and external factors in counterfactual thoughts and emotions such as guilt and shame. The study involved 160 drivers, of whom 54 were categorized as repeat offender drivers while 106 drivers attended courses for advanced professional driving licenses. The participants drove along a route in a driving simulator, which had been previously adjusted for the difficulty to generate a perception of high or low control. Based on the outcome obtained by the participants in this stage, each driver had to report which resources they required to improve their outcomes. Different factor ANOVAs were used to analyze our findings. The results indicated that optimistic offenders, unlike other groups (i.e., optimistic non-offender and pessimistic non-offender), thought that their results could have been better if external factors had been present (i.e., upward counterfactuals), both under conditions of high and low control. They believed their results would have been worse had it not been for their internal resources (i.e., downward counterfactuals), especially under conditions of low control. Concerning emotions of guilt and shame, offender optimists had the lowest values in both conditions compared with the other groups. We may contend that optimistic offender drivers thought they could have obtained better outcomes if external factors had been involved. In the low control condition, they justified that if it were not for such internal skills, their results could have been worse. When they generated such thoughts, the emotions of guilt and shame were minimal.

## Introduction

Most traffic accidents are the result of risky behaviors performed by drivers. Cognitive-motivational theoretical models of traffic psychology analyze how drivers perceive risk and make decisions. Experimental evidence suggests that a relatively stable cognitive bias tends to exist among drivers, leading them to determine how likely they will be to have an accident (Harré et al., 2005). In some cases, the perception of risk of the driver may be connected to the situation of assumed risk, causing the driver to behave cautiously, judiciously, etc., as predicted by the so-called Zero-Risk Model (Näätänen and Summala, 1974). In other cases, when faced with a challenging situation, drivers may adopt driving skills focused on elements such as technique, ability, and mastery. Such skills explain the subjective perception the driver maintains regarding the perceived risk of being involved in a road accident and the perceived level of risk he or she is willing to assume, as outlined in Risk Homeostasis Theory (Wilde, 1982). As contended by Task Difficulty Allostasis (Fuller, 2011), this sense of risk may vary, impacting the adjustment to the perceived difficulty of the task and the prior driving skills and experience of each individual as the difficulty perceived increases.

The scientific literature encompasses numerous studies that focus on risky driving, which has been associated with different factors: tailgating, driving under the influence of drugs or alcohol, distractions, road rage, speeding, drowsy driving, and the non-use of safety belts (Dula et al., 2011; Mãirean and Havârneanu, 2018). There are fewer studies that analyze the cognitions generated by the subject regarding the “why and how” things happen. Malle and Tate (2006), for example, studied both the reasoned explanations provided by drivers regarding their intentional behavior and how the drivers believed they could achieve the desired results. Our focus here is on a line of research analyzing alternative thoughts generated after the event has taken place or after the result has been previously obtained. This type of thinking is known as “counterfactual thinking” (Roese and Morrison, 2009; Byrne, 2016; Epstude, 2018).

These types of thoughts are important because they imagine changing certain aspects of the mental representation of reality. The cognitive process developed is the creation of imagined alternatives and a process of comparison between these alternatives and the actual results obtained (Byrne, 2016). Therefore, the focus of counterfactual thinking is on thoughts related to what might have been, or how the past might have been different had certain aspects been different (Smallman and Summerville, 2018). Counterfactual thinking is represented as conditional propositions, which contain an “if” antecedent followed by a “then” consequent. This type of conditional structure commonly reflects a causal inference: “If I had been more cautious, (then) the crash could have been avoided” (that is, driving more cautiously is enough to avoid a collision). In this case, neither the antecedent action nor the consequent result happened, so counterfactual thinking focuses on what could have been, not on what happened. We can contemplate real relationships in this causal inference that the subject regards as very probable (i.e., if I drive prudently, then I avoid a collision with other vehicles). These types of thoughts, therefore, are meant to explain the past while simultaneously preparing for the future. They involve various associations, including causal associations, and affect intentions as well as decisions. Counterfactual thoughts are, therefore, precursors in the formation of intentions to carry out future actions.

Different counterfactuals can be contemplated, depending on the following dimensions. If we consider a “direction,” two types of counterfactuals can be distinguished: upward, which reflects mental simulations where the possible results are better than those obtained in reality; and downward, which is related to mental simulations where the possible results are worse than those obtained in reality. Conversely, if we consider the “structure” dimension, a counterfactual thought could be additive, as when an event could or should have transpired [e.g., if I had respected the zebra crossing, (then) I would not have hit the pedestrian], as opposed to when the event should not have occurred (e.g., if I had not gone so fast, I wouldn’t have crashed); it would reflect a subtractive counterfactual. Finally, there is the “focus” dimension. A counterfactual thought could focus on oneself or other people or circumstances. This dimension could also focus on controllable vs. uncontrollable factors, although the factors in this dimension are typically covaried. One possibility would be that self-focused counterfactuals were controllable (e.g., If only I had paid more attention …) and other focused counterfactuals were uncontrollable (e.g., If it had not been raining…). Another possibility would be that self-focused counterfactuals were uncontrollable (e.g., If I were younger …) and other-focused counterfactuals were controllable (e.g., If there had been more surveillance on the highway …) (Epstude, 2018; Smallman and Summerville, 2018).

The connection between counterfactual thoughts and emotions is established under the framework of “what could have been,” compared to the current moment (Suls and Wheeler, 2000; Mandel, 2003; Markman et al., 2008; Epstude and Roese, 2011; Tasso et al., 2017; Epstude, 2018). Seta et al. (2015) reported that when alternative worlds are better than the results experienced, emotion and cognition may be more closely linked to alternative realities than to the results obtained. Nevertheless, as a general rule, when the reality is compared with its counterfactual alternative, the emotions can be amplified (Giguère et al., 2014; Byrne, 2016). If the discrepancies between the result obtained and the desired result are minor, positive emotions may appear (e.g., relief, satisfaction, and sympathy). Conversely, greater discrepancies may result in the appearance of negative emotions (e.g., regret, guilt, and shame). In particular, under the latter condition, individuals may demonstrate repetitive, intrusive, and negative cognitions, referred to as ruminative thoughts, which may amplify negative affective states, making it difficult to solve problems (Spellman and Mandel, 1999; Wade et al., 2008; Cavalera et al., 2018).

Specifically in the case of upward counterfactual thoughts when the antecedent focuses on a personal choice (e.g., “If I had not drunk, I would have avoided road collisions”), moral emotions of shame or guilt appear if the established criteria and norms have not been met in the performance of the appropriate behaviors (Zeelenberg et al., 1998; Etxebarria, 2003; Tangney et al., 2007; Giguère et al., 2014). However, these moral emotions have their differences. Guilt appears when someone perceives that he or she is responsible for the failure in a situation considered controllable (Tangney and Dearing, 2003). The emotion of guilt facilitates the appearance of reparative behaviors to modify the result obtained (Tangney et al., 2014). Instead, when someone experiences shame, he or she attributes this to his or her failures, and not to his or her behavior in situations where there is no perceived control (Tracy and Robins, 2007). When a person experiences shame, he or she forms negative judgments about his or her abilities, generating a desire to flee and disappear (Tangney et al., 2014). Thus, a negative evaluation of own behavior and feelings of remorse or regret may be associated with guilt, while a negative evaluation of the self and feelings of helplessness or insignificance may be associated with shame (Tangney and Dearing, 2003; Schmader and Lickel, 2006; Tracy and Robins, 2007).

Different authors (Niedenthal et al., 1994; Roese and Olson, 1997) have studied the role of counterfactual thinking in reference to guilt and shame. They argue that guilt is usually associated with upward counterfactual thoughts in the form of “If I hadn’t overtaken the car, I would not have crashed,” where the alternative picks up a specific behavior, as a cause of the unwanted result. Shame, on the other hand, emphasizes the cause in the proposed alternatives, questioning the abilities of the person and focusing on the need for a change in personal behavior to undo the result. One upward counterfactual thought associated with shame, where the person has no ability, could be “If I weren’t such a distracted person, I would not have had this crash.” Finally, it should be noted that downward counterfactual thinking is linked to feelings such as satisfaction or relief. This type of thinking can fulfill a function of mood repair, as it tends to make the person feel better (Roese and Olson, 1995; Sanna, 1998; Sanna et al., 2001).

The process of self-regulation is consistent with this approach. It is triggered when an individual considers his or her present status and compares it to a more desirable one in which behavior and emotion reflect feedback control (Carver and Scheier, 2016). In their theory of the self-regulation of behavior, Scheier and Carver (1985) contend that optimists strive to achieve a desirable outcome, which they believe themselves capable of attaining. In contrast, pessimists consider the outcomes to be unattainable and will, consequently, either desist or fail to commit to the actions required to attain the target outcome. Sundry studies have focused on analyzing how different future scenarios are constructed in the minds of optimists and pessimists (Sharot, 2011; Garrett and Sharot, 2017). It is important to consider the difference between optimism and pessimism as cognitive expectations associated with future events, and counterfactual thinking as a cognitive process involving the imagination of alternative outcomes to past events.

This leads us to the focus in this study on the conception of optimistic vs. pessimistic expectations, as proposed by Carver and Scheier (2016), which indicates that dispositional optimistic expectations generally focus on the results to be obtained. This study will consider a fundamental aspect of the human behavior factor in road safety, namely, the personality traits of the drivers and their disposition, in particular, for optimism and pessimism (del Valle and Mateos, 2008; del Valle, 2019; del Valle and Matus, 2019). Similar to the study of Giguère et al. (2014), we believe that counterfactual thoughts are decisive in the study of the self-regulatory functions of guilt and shame. The consideration that counterfactual thoughts create different scenarios or alternatives in the achievement of the proposed objectives can provide insight into the causal attribution that subjects make of the result of the current situation. Different authors (Markman et al., 1995; Roese and Olson, 1997; Nasco and Marsh, 1999) point out, after obtaining a negative result, upward counterfactual thoughts are more frequent than downward counterfactuals because the upward counterfactuals reflect what the participants could have done to obtain better results. The functionality of downward counterfactual thoughts is related to the justification of the results obtained since they imagine it could have been worse.

A determining variable that mediates between optimistic and pessimistic expectations and counterfactual thoughts is the perceived control that drivers believe they have because it primarily functions as a risk moderator (Girotto et al., 1991; Helweg-Larsen and Shepperd, 2001; Klein and Helweg-Larsen, 2002). Several studies have found that drivers who overestimate their driving skills and their perceived degree of control are more inclined to drive dangerously while maintaining a more optimistic view on the risk of having an accident (Sümer et al., 2006; Fernandes et al., 2007; Harre and Sibley, 2007). The degree of control or perceived controllability is a perception of an individual of his or her capacity, available resources, or opportunities to obtain positive results or to avoid negative ones through his or her behavior (Thompson, 1981). Similar to the study of Epstude and Roese (2008), we contend that people exaggerate the personal control they believe they have over a given situation. People who generate counterfactual thoughts tend to report factors that they believe could be manipulated, controlled, or altered in some way (Byrne, 2016). There are events in daily life that are controllable (e.g., use of the seat belt) and others that are uncontrollable (e.g., traffic jams). When people generate counterfactual thoughts, they tend to imagine how the results would have been different if they had behaved differently, albeit, to a greater extent, in situations, they believe they control (Girotto et al., 1991). These controllable situations have a greater likelihood of eliciting upward (i.e., “it could have been better”) than downward counterfactuals (Roese and Olson, 1995), while upward counterfactuals enhance retrospective control perceptions (McMullen et al., 1995; Nasco and Marsh, 1999). For example, Davis et al. (1995) have noted that the “controllability effect” appears in traffic accidents involving the loss of a loved one. Drivers that have not caused the accident focus on alternatives to their behavior rather than on the alternative actions of the driver who caused the accident (“If we had not gone by car that day, we would not have had the accident”).

In the context of driving, perceived high control can inflate estimation of the drivers of their ability, whereby both the optimism and the perceived controllability of the event maintain a close relationship (Harris, 1996; White et al., 2004). Optimistic biases of an individual are expressed in their perception of personal risk, so that, in the event of an accident, the individual tends to attribute it more to external factors (e.g., rain, a blowout), as opposed to internal factors related to driving (Harré et al., 2005). Optimistic offender drivers in their driving habits make the decision to carry out risky behaviors, even knowing that these involve high probabilities of risk (e.g., increasing speed, crossing an amber traffic light, not respecting the distance of security, or checking the mobile). McKenna (1993) indicated that people believe themselves to be less likely to suffer a road accident than others when they are driving (i.e., personal control). When they are passengers, the chances of having an accident are no different than those of other people. The illusion of control is what prompts them to attribute accident-free driving to their ability rather than the effect of external factors (Hammond and Horswill, 2002).

The last variable is related to drivers who have committed traffic violations, such as driving over the alcohol limit, etc. When these offending drivers lose points on their licenses, they must attend reeducation courses to recover some or all of the points lost. In Spain, these courses are known as “intervention, awareness, and road reeducation courses in the license points system, the Directorate General for Traffic (DGT—Dirección General de Tráfico).” Various studies have been conducted in Spain with drivers who have lost their license (Cuervo and Villanueva, 2015; Valero et al., 2017; Faílde et al., 2018; Padilla et al., 2018; Martí-Belda et al., 2019). We have conducted previous studies focusing on this group of offender drivers, and we have analyzed a type of anticipatory thinking, called pre-factual thoughts. del Valle (2019) has obtained a cognitive profile of optimistic offender drivers, indicating that they believe themselves to be more likely to achieve their desired outcomes regardless of the driving conditions, considering themselves to be more skillful drivers than their peers. In another study, del Valle and Matus (2019) reported similar findings, indicating that optimistic offender drivers consider themselves more likely to achieve their desired outcomes and do not question their skills or resources. They believe their results would be even better were they assisted by external factors. However, this group does not record better results than the other groups, so this thinking is not realistic.

There are no conclusive studies in the scientific literature that analyze alternative thoughts related to “what could have been” (i.e., counterfactual thoughts) and the emotions of guilt and shame in optimistic offender drivers. Therefore, this study aimed to analyze the extent to which the controllability of the situational context in a simulated driving task performed by optimistic offender drivers who attend reeducation courses influenced their use of internal and external factors in counterfactual thoughts and negative emotions (i.e., guilt and shame). As regards this objective, we consider the extent to which optimistic offender drivers, under conditions of induced control, are different from non-offenders drivers due to the effects of the internal and external factors on counterfactual thinking and in the experiences of guilt and shame.

## Materials and Methods

### Participants

One hundred and eighty-two voluntary drivers anonymously took part in the study. Twenty-two of the volunteers were disqualified: 20 because they did not meet the inclusion criteria in the study groups according to optimistic vs. pessimistic expectations (i.e., four were dispositional pessimistic offenders, six were defensive pessimistic offenders, and 10 were defensive pessimistic non-offenders). Two optimistic offenders were discarded for not completing the study correctly and/or for not following the instructions throughout the different stages. Consequently, the final sample consisted of 160 participants.

The study involved three groups. The first criterion to establish the group study was based on driving offenses, offender (*N* = 54), and non-offender (*N* = 106). The offender group was formed by drivers who, with the purpose of regaining their licenses or the points deducted for their repeated offenses, were attending courses on road intervention, awareness, and rehabilitation as stipulated within the framework of the points-based license system applied by the Directorate General for Traffic (DGT) in Spain. The non-offender group consisted of people who were either attending courses for professional drivers, known as Certificate of Professional Proficiency (CAP- Certificado de Aptitud Profesional), or attending courses for obtaining another type of license. None of the drivers in the non-offender group had previously attended a course to recover points, nor had they lost any points over the past 2 years. None of the drivers in the offender group had previously attended an advanced driving course for professional drivers. The data were gathered from various driving schools in the city of Salamanca (Spain).

### Sample Descriptions

The participants in the study were primarily male (76.3%), whose average age was 37.5, who were single (51.9%), and who had completed primary (30%) or higher (32.5%) education. Table 1 shows the statistics of the demographic characteristics and measures among the proposed groups.

**TABLE 1:** Statistics of demographic characteristics and measures between Study Group.

	Optimistic offender *n* (%)	Optimistic non-offender *n* (%)	Pessimistic non-offender *n* (%)
**Sex**			
Male	41 (75.9)	38 (70.4)	43 (82.7)
Female	13 (24.1)	16 (29.6)	9 (17.3)
	54 (100)	54 (100)	52 (100)
	*M*(*SD*)	*M*(*SD*)	*M*(*SD*)
Age	37.22 (10.11)	36.07 (11.56)	39.27 (10.94)
**Marital status**			
Single	29 (53.7)	27 (50.0)	27 (51.9)
Married	12 (22.2)	17 (31.5)	14 (26.9)
Divorced/separated	13 (24.1)	10 (18.5)	11 (21.2)
**Level of education**			
Primary	13 (22.2)	17 (31.5)	19 (36.5)
Secondary	20 (37.0)	16 (29.6)	06 (11.5)
3-year degree	4 (07.4)	03 (05.6)	10 (19.2)
5-year degree	17 (31.5)	18 (33.3)	17 (32.7)
LOT-R	34.59 (3.02)	30.35 (4.51)	19.67 (4.18)
Positive PANAS	33.20 (7.14)	30.91 (5.78)	16.10 (2.73)
Negative PANAS	12.63 (4.65)	14.48 (2.74)	34.19 (3.95)
Guilt PFQ-2	0.40 (0.21)	1.56 (0.55)	1.40 (0.51)
Shame PFQ-2	0.43 (0.23)	1.25 (0.45)	1.23 (0.33)

To confirm whether the groups were identical, chi-square tests were conducted in the case of categorical variables, while one ANOVA was conducted for independent samples in the case of quantitative samples. To analyze possible interactions between groups, a Bonferroni correction was applied. No significant differences were found in sex χ^2^_2_ = 2.23, *p* = 0.329, or in marital status χ^2^_4_ = 1.32, *p* = 0.857. Regarding the level of education, optimistic offenders were found to have received lower scores in primary school, more scores in secondary school, and fewer scores in a 3-year-degree program than the pessimistic non-offender (χ^2^_6_ = 13.77, *p* = 0.032). No significant differences were found in age [*F*(2,157) = 1.17, *p* = 0.314]; no significant differences between groups were discovered.

### Variables and Measurement

#### Mood States

The Spanish version of the *Positive and Negative Affect Schedule* (PANAS; Watson et al., 1988; Sandin et al., 1999) is a questionnaire used to measure the general emotional state of an individual according to responses provided. The questionnaire includes 20 items with 10 negative and 10 positive affects, which are scored on a 5-point Likert scale of 1 (very slightly or not at all) to 5 (extremely). In our study, the Cronbach’s α for the negative and positive subscales are 0.83 and 0.85, respectively.

*Personal feelings questionnaire* (PFQ-2; Harder and Zalma, 1990; translated into Spanish by Popa, 2013) was used to assess guilt and shame. This is a questionnaire consisting of 22 items, with a Likert-type response format, where 0 means “*never*” and 4 “*many times*,” depending on how often the person experiences the feelings reflected in each item. Of the 22 feelings, only those that measure feelings of guilt (e.g., remorse, regret, worry about hurting someone…) and feelings of shame (e.g., stupidity, embarrassment, humiliation…) are scoring, specifically 16 items. The remaining six items have the function of concealing the purpose of the questionnaire and are not used to obtain the results. In our study, the internal consistency of the Shame subscale and the Guilt subscale was found to be adequate (Cronbach’s α = 0.78 and 0.77, respectively).

#### Optimistic and Pessimistic Expectations

The Spanish version of the *Life Orientation Test-Revised* (LOT-R; Scheier et al., 1994; Perczek et al., 2000) is a tool used to measure optimism and pessimism as they relate to the personality disposition of an individual. The questionnaire comprises six items and generates a continuous distribution of scores. Respondents answer each question on a 5-point Likert scale (1 = *I completely agree*, 5 = *I completely disagree*). Cronbach’s α for pessimism and optimism was found to be 0.80 and 0.86, respectively.

The Spanish version of the *Optimism-Pessimism Questionnaire* (OPQ; Norem and Cantor, 1986; Fernández and Bermúdez, 1999) aims to estimate the personality disposition of the respondent on a dimension of optimism and pessimism. The questionnaire is a 9-item measure and uses a 10-point Likert scale of 1 (*I completely disagree*) *to* 11 (*I completely agree*) for three subscales: *Optimistic*, *pessimistic*, and *referring to past performance*, comprising four, four, and one item, respectively. The internal consistency for the optimism scale was 0.84, while the result for the defensive pessimism scale was 0.83.

#### Counterfactual Thinking

We followed the procedure used in previous research to assess counterfactual thoughts (Petrocelli et al., 2011; Ferrante et al., 2013; Scholl and Sassenberg, 2014; Roese et al., 2017). In the introduction, we noted that counterfactual thinking involves mental simulations of different alternatives that could have been undertaken to achieve a different result than the one obtained. In the instructions in assessing the counterfactual thoughts, we told the participants that we were interested in knowing which alternative antecedents (i.e., behaviors, circumstances, etc.) they believed were necessary for achieving the desired result or avoiding an unwanted one.

Three judges agreed on the process of analyzing and coding the different counterfactual thoughts. Each sentence included two interactive components: the “*If*…” antecedent to indicate an alternative action or condition, which could have but did not occur; and the “*Then*…” consequent to identify a desired alternative result, which could have been, but was not, obtained. Each counterfactual thought was coded according to its direction (upward or downward) and whether the alternative antecedents included external or internal resources.

The judges used upward counterfactuals to code whether the possible results were better than those obtained in reality. The thought was coded as downward counterfactual if the possible results were worse than those obtained in reality. Similarly, the type of resource (internal or external) was used to code counterfactual thinking. For example, judges should code the statement “If I had driven more cautiously, then the accident wouldn’t have occurred” as upward counterfactual and internal factors. In the case of “If the tire had burst, then I could have had an accident,” the judges should code it as downward counterfactual and external factors. By following this procedure, “upward counterfactual” and “downward counterfactual” can each result in a single score, reflecting the resources used by the participant.

### Experimental Conditions

In order to measure the driving skills and the different cognitive capabilities of each of the participants, they were asked to drive the “Advanced Road Safety Education” module in the DriveSim simulator. Two types of tasks were created for the experimental design of the study. The situational control was adjusted to allow for either low or high control. In addition to using the instructions provided with each task, we also selected different routes on the traffic simulator. For each of the tasks, we modified the instructions. In the low control task, the instructions referred to difficulty, whereas, in the high control task, they referred to manageable aspects.

The level of difficulty on the traffic simulator was adjustable, allowing us to induce low or high situational controllability. Low controllability was induced with a more complex and aggressive model, which involved difficult weather conditions, such as rain or fog, the presence of other drivers, and more aggressive traffic. These conditions resulted in more aggressive driving such as abrupt acceleration and braking. High situational controllability was induced with a less complex model, which involved pleasant dry and sunny weather conditions, and no additional drivers on the road. Upon completing the routes, the simulator showed the participants which mistakes they had made.

### Procedure

The study met the approval of the Bioethics Committee of the University of Salamanca. We developed a software program to provide all the data and enable us to control the sample selection processes and the application of the different phases of the study. During the first phase of the study, the participants were told that their cognitive capabilities and driving skills would be measured and analyzed. They were required to sign a letter of informed consent (model CBEA1) adapted to the characteristics of the project, prior to participating in the study, and their sociodemographic data were collected. The emotion (i.e., PANAS) and expectations questionnaire (i.e., LOT-R and OPQ) were administered in the second stage when the sample selection criteria were applied.

The participants were assigned to the groups based on the scores they obtained in the LOT and the OPQ. We followed the procedure carried out by Fernández and Bermúdez (2000) to establish the optimistic vs. pessimistic groups. Different authors (Cantor and Norem, 1989; Elliot and Church, 2003) found that, within the established continuum between pessimism and optimism, there was a group called defensive pessimism. Considering that prior studies (Sanna, 1998; Norem and Chang, 2002; del Valle and Mateos, 2008) have shown that defensive pessimists have a differential profile in terms of emotional state and factual thoughts, we have discarded such cases from our analysis to define the study groups more reliably. The defensive pessimist group and the dispositional pessimism group, would declare pessimistic expectations about the future and focus on possible difficulties and negative results; moreover, they would display a lower perceived control and high levels of anxiety (Showers, 1992). As opposed to dispositional pessimists, defensive pessimists can recall successes achieved in the past about the same task-related problems and difficulties. Additionally, as with optimists, defensive pessimists are capable of taking on any tasks necessary to avoid the anticipated negative result.

In an effort to more precisely define the study groups, optimistic and dispositional pessimistic participants had coincided in their respective groups in both questionnaires, a procedure followed in previous experiments (Cantor and Norem, 1989; Elliot and Church, 2003). We considered both the item of past performance and the subscale of pessimism in the OPQ. This allowed us to distinguish between defensive pessimists, who reported a higher past performance, as indicated by a score of seven or more, and dispositional pessimists, who acknowledge previous negative behavior, as indicated by a score of six or less on the response scale.

Excluded individuals (i.e., defensive pessimists and dispositional pessimist offenders) performed a shorter task, which was not included in the study. The remaining members of the study sample were pseudo-randomly assigned one of the two conditions proposed (i.e., high control or low control). The participants were unaware of the existence of both conditions. The results of the questionnaire were used to determine the degree of optimism and pessimism of each participant. Using this information, the computer program assigned the participants to one of the conditions. We hoped that a similar number of participants would comprise each group. During the third phase, the participants were required to complete the assigned task. Upon completing the route, the participants were able to view the mistakes they had made and discuss them with the researcher.

Counterfactual thoughts were assessed during the next stage. The program software displayed a grammatical structure of a conditional subordinate clause to the participants, who then had to write the circumstances under which the action of the main sentence took place “If., then.” In the first part of the structure (If), the participants were asked to fill in the gaps using internal or external factors that would have been necessary to achieve the desired results of the task or avoid the undesired ones. When each of the sentences had been completed, the participant had to choose those items (i.e., guilt and shame) in the PFQ-2 questionnaire that best reflected their emotional state according to the chosen phrase.

### Experimental Design

The experimental design of the study considered the following variables: group study (optimistic offenders, optimistic non-offenders, and pessimistic non-offenders) and task condition (high control vs. low control), with two dependent variable resources in upward and downward counterfactual thinking.

We have already specifically noted that the dependent variables reflect the resources (internal and external) used by the study participants when they generate counterfactual thoughts. The degree of agreement between raters on the assignment of a categorical variable was measured (Kappa = 0.71, *p* < 0.001). A single score was recorded for each type (upward and downward) of counterfactual thinking. The score recorded for external factors was subtracted from the score for internal factors, which could result in a negative score if the participant mentioned more internal than external factors. Another dependent variable involved emotions (i.e., guilt vs. shame). In all cases, the tests were subjected *a posteriori* to a Bonferroni correction to analyze the interactions.

## Results

First, different ANOVAs were used to analyze whether the established groups presented significant differences in the LOT and the PANAS questionnaires. Second, additional ANOVAs were carried out to analyze whether the study groups presented differences in the total number of counterfactual thoughts and to analyze the mistakes made during the simulator task. Third, a correlation analysis was applied to analyze the existence of significant relationships between errors committed and negative emotions (i.e., guilt and shame). Fourth, to analyze whether the study groups in the proposed conditions presented differences in two variables—counterfactual factor (upward and downward) and negative emotions (i.e., guilt and shame)—two multivariate repeated measures analysis of variance (MRM-ANOVA) were carried out.

The SPSS v.25 Statistics software was used to perform the analysis. A level of significantly lower than α = 0.05 was the reference established to consider the differences significant. When significant differences were present, all the analyses calculated the size effect.

### Preliminary Results

As regards the questionnaires administered, the scores of the questionnaires in each group were normally distributed. To establish whether the groups presented differences in the LOT-R, we carried out a one-way ANOVA and *post hoc* test for the LOT-R score for the groups. Statistically significant differences were noted in the LOT [*F*(2,157) = 199.89, *p* < 0.001] between all groups. To establish whether the groups presented differences in the positive PANAS and negative PANAS, we carried out two one-way ANOVAs and a *post hoc* test for positive PANAS and negative PANAS scores for the groups. We also found differences in the positive PANAS [*F*(2,63) = 147.01, *p* < 0.001] and the negative PANAS [*F*(2,157) = 441.19, *p* < 0.001] scores, in addition to significant differences between the pessimistic non-offender and the optimistic offender (*p* < 0.001) and optimistic non-offender (*p* < 0.001) groups (as shown in Table 1). However, no significant differences were found between optimistic non-offenders and optimistic offenders.

According to the total number of counterfactual thoughts, to establish whether there were differences in the total number of counterfactual thoughts between groups and conditions, we carried out an ANOVA on the total number of counterfactual thoughts. We did not find differences according to the groups [*F*(2,154) = 2.14, *p* = 0.121], nor under the conditions [*F*(2,154) = 0.78, *p* = 0.379], and we found differences under the interaction Groups × Conditions [*F*(2,154) = 42.36, *p* < 0.001] (as shown in Table 2).

**TABLE 2:** Descriptive statistics in counterfactual thinking as a function of Task Condition and Study Group.

	Task condition	
	High control	Low control
Study group	M (SD)	M (SD)
Optimistic offender	2.25 (1.43)	5.00 (1.33)
Optimistic non-offender	3.56 (1.28)	3.78 (1.28)
Pessimistic non-offender	4.33 (1.88)	1.96 (2.27)

Regarding mistakes made during the task with the simulator, we carried out an ANOVA on total number of mistakes. We did not find differences between groups and conditions [*F*(2,154) = 1.39, *p* = 0.252], nor between groups [*F*(2,154) = 2.03, *p* = 0.134], but we have found that, under the condition of high control, there were significantly fewer mistakes than under the condition of low control [*F*(2,154) = 129.80, *p* < 0.001, eta^2^ = 0.457] (see Table 3).

**TABLE 3:** Difference in means and standard error between the optimistic offender and the optimistic non-offender and pessimistic non-offender groups under task condition for the following variables: mistake, upward counterfactuals, downward counterfactuals, guilt, and shame.

Variables	Study group	Task condition	
		High control	Low control
		M (SD)	M (SD)
Mistake	Opt-offe vs Opt-non-offe	−0.135(1.393)	−0.413(1.419)
	Opt-offe vs Pess-non-offe	3.124 (1.393)	0.086 (1.447)
	Opt-non-offe vs Pess-non-offe	3.259 (1.406)	0.499 (1.433)
Upward counterfactuals	Opt-offe vs Opt-non-offe	−1.511**(0.314)	−2.734**(0.319)
	Opt-offe vs Pess-non-offe	0.881*(0.314)	2.888*(0.326)
	Opt-non-offe vs Pess-non-offe	−0.630(0.316)	0.154 (0.323)
Downward counterfactuals	Opt-offe vs Opt-non-offe	1.530**(0.226)	−1.266**(0.230)
	Opt-offe vs Pess-non-offe	0.049 (0.226)	−2.192**(0.234)
	Opt-non-offe vs Pess-non-offe	−1.481*(0.228)	−0.926**(0.232)
Guilt	Opt-offe vs Opt-non-offe	−0.763**(0.092)	−1.574**(0.094)
	Opt-offe vs Pess-non-offe	−1.312**(0.092)	−0.669**(0.096)
	Opt-non-offe vs Pess-non-offe	−0.549**(0.093)	−0.230**(0.095)
Shame	Opt-offe vs Opt-non-offe	−0.316*(0.063)	1.325**(0.064)
	Opt-offe vs Pess-non-offe	−0.546**(0.063)	−1.071**(0.065)
	Opt-non-offe vs Pess-non-offe	0.905*(0.063)	0.254**(0.065)

*p < 0.05 and **p < 0.001. Opt-offe, Optimistic Offender; Opt-non-offe, Optimistic Non-Offender; Pess-non-offe, Pessimistic Non-Offender.

When we analyzed whether the number of mistakes occurring during the driving task was related to the levels of shame and guilt, between groups and conditions, we found that only the optimistic offender group under the condition of low control showed relationships between mistakes and shame (rx = 0.515 *p* = 0.007).

### Counterfactual Thinking

We performed a MRM-ANOVA, which involved two factors. The first was a between-subjects factor based on “Group Study” (optimistic offender, optimistic non-offender, and pessimistic non-offender), and the second was a within-subjects factor based on “Task Conditions” (low control vs. high control) as dependent variable resources in the counterfactual factor (upward and downward).

Analyses revealed a significant interaction between Group Study × Task conditions × Counterfactual thoughts was significant [*F*(2,154) = 33.91, *p* < 0.001, eta^2^ = 0.31]. Group Study × Counterfactual thoughts [*F*(2,154) = 53.59, *p* < 0.001, eta^2^ = 0.41]. Task condition × Counterfactual thoughts [*F*(1,154) = 1.508, *p* = ns]. Bonferroni adjusted *post hoc* analyses tests revealed that significant differences were found in “high control” condition in upward counterfactuals [*F*(2,154) = 11.737, *p* < 0.001, eta^2^ = 0.13], but no statistically significant differences were found among all groups. While significant differences were found between groups comprising optimistic offenders and optimistic non-offenders (*p* < 0.001) and pessimistic non-offenders (*p* = 0.017), no statistically significant differences were found between optimistic non-offenders and pessimistic non-offenders (*p* = 0.145). Moreover, Bonferroni-adjusted *post hoc* analyses revealed differences in “high control” condition in downward counterfactuals [*F*(2,154) = 29.404, *p* < 0.001, eta^2^ = 0.28]. Significant differences were found between groups comprising optimistic offenders and optimistic non-offenders (*p* < 0.001) and optimistic non-offenders and pessimistic non-offenders (*p* < 0.001), but not significant differences were shown between optimistic offenders and pessimistic non-offenders (*p* = 0.100); (as shown in Table 3).

In “low control” condition in upward counterfactuals, Bonferroni-adjusted *post hoc* test showed differences [*F*(2,154) = 50.679, *p* < 0.001, eta^2^ = 0.40]. While statistically significant differences were found between the groups comprising optimistic offenders and optimistic non-offenders (*p* < 0.001) and pessimistic non-offenders (*p* = 0.017), none were found between the optimistic non-offenders and pessimistic non-offenders (*p* = 0.100). Bonferroni-adjusted *post hoc* analyses revealed differences in “low control” condition in downward counterfactuals [*F*(2,154) = 44.258, *p* < 0.001, eta^2^ = 0.36]. Statistically significant differences were found between all the groups: optimistic offenders and optimistic non-offenders (*p* < 0.001) and pessimistic non-offenders (*p* < 0.001), significant differences were found between the optimistic non-offenders and pessimistic non-offenders (*p* < 0.001) (see Table 3).

### Emotion

A two-way MRM-ANOVA was conducted to determine the effects of Group Study and Control Task in Negative Emotions factor (guilty and shame).

Analyses revealed a significant interaction between Group Study × Task conditions × Negative Emotions was significant [*F*(2,154) = 19.51, *p* < 0.001, eta^2^ = 0.20]. Group Study × Negative Emotions [*F*(2,154) = 2.10, *p* = ns]. Task condition × Negative Emotions [*F*(1,154) = 37.73, *p* < 001, eta^2^ = 0.20]. After performing the Bonferroni-adjusted *post hoc* analyses tests, we found differences in the “high control” condition in guilt [*F*(2,154) = 81.26, *p* < 0.001, eta^2^ = 0.51], analyses showed significant differences between all groups (*p* < 0.001). Additionally, Bonferroni-adjusted *post hoc* analyses revealed differences in “high control” condition in shame [*F*(2,154) = 16.99, *p* < 0.001, eta^2^ = 0.18]. Significant differences were found among all the groups: optimistic offenders and optimistic non-offenders (*p* = 0.004) and pessimistic non-offenders (*p* > 0.001), and significant differences were shown between the optimistic non-offenders and pessimistic non-offenders (*p* = 0.035). In “low control” condition in guilt, Bonferroni-adjusted *post hoc* test showed there were significant differences [*F*(2,154) = 114.55, *p* < 0.001, eta^2^ = 0.60], and analyses showed statistically significant differences between all groups (*p* < 0.001). Bonferroni-adjusted *post hoc* analyses revealed that there were significant differences in “low control” condition in shame [*F*(2,154) = 156.33, *p* < 0.001, eta^2^ = 0.67]. Significant differences were found among all the groups (*p* < 0.001) (as shown in Table 3).

## Discussion

We have analyzed to what extent the optimistic offender drivers, under conditions of induced control, differed from non-offender drivers in the effect of internal and external factors on counterfactual thinking and emotions (i.e., guilt and shame). It was found that optimistic offenders in both high- and low-control conditions used upward counterfactuals linked to external resources more than the other groups. When these participants generated these thoughts, they indicated that they could have obtained better results if there had been external factors involved. In relation to the downward counterfactuals, these participants had a different profile, depending on the control conditions. In a low-control condition, they were linked to a greater extent with internal factors. The belief that things could have been worse if they had not done something highlights protective functionality on an emotional level. With respect to emotions, we observed that this group recorded the lowest scores for negative emotions (i.e., guilt and shame) in both conditions and compared to the rest.

Consistent with previous studies (del Valle, 2019; del Valle and Matus, 2019), optimistic offenders overestimated their overall level of success. We believe that counterfactual thinking can have a dysfunctional implication. When drivers generate upward counterfactual thinking in explaining their failures or unwanted results, they overestimate their abilities (Petrocelli et al., 2012). Different studies have indicated how optimistic offender drivers have a greater tendency to qualify as more skilled compared with other drivers (Dogan et al., 2012; Horswill et al., 2013; Stephens and Ohtsuka, 2014; Mãirean and Havârneanu, 2018). These participants focus on external aspects of the situation (“If it hadn’t snowed, then I could have avoided the accident”) to justify their unwanted results, as noted by Girotto et al. (2007). Most of the causal attributions involved in upward counterfactuals tend to diminish the extent of the problem (i.e., if you had only had more time.) instead of considering other possibilities (i.e., inadequate knowledge or a misunderstanding of the problem) (Sherman and McConnell, 1995).

We contend that it is precisely this self-assessment that these drivers make of the abilities that lead them to ignore, or at least underestimate, the negative feedback provided by the environment (Allison et al., 1996; Petrocelli et al., 2012). The fact that these drivers ignore what happened, thinking about what “could have been,” may explain why these drivers have the lowest values in negative emotions (i.e., guilt and shame), both in low- and high-control conditions, as we will be commenting on in due course.

In relation to downward counterfactuals, the optimistic offenders in the low-control condition note how the results obtained could have been worse if they had not done something. These drivers also used this thinking to justify the mistakes made. In addition, these thoughts could have an affective function, helping people to feel better, as they reduce the potentially negative impact of considering an unattained result (Epstude and Roese, 2008; Sweeny and Vohs, 2012; Byrne, 2016). Similar to the study of Smallman and Summerville (2018), we think that one of the functions of counterfactuals is to provide reasons for poor performance, facilitating different excuses. This type of counterfactual implicitly denies the existence of any possibility of improvement, and it can reduce the motivation to change and improve. In these cases, counterfactuals can justify self-esteem of an individual while concurrently reducing the motivation for successive improvement (McCrea, 2008).

As mentioned earlier, optimistic offenders recorded the lowest values of negative emotions (i.e., guilt and shame). Tangney and Dearing (2003) have commented that emotions such as guilt depend on the negative judgment of a person of his or her action. This emotion tends to appear in situations in which a failure is perceived; there is a perception of controllability in his or her actions, and, therefore, the driver is attributed internal responsibility for it (e.g., “If I had not had that drink, the collision would have been avoided”). Some authors (Fedewa et al., 2005) have commented that guilt can encourage actions to amend the result generated; on the one hand, these drivers do not feel guilt, and, on the other, they attribute responsibility for the result to external aspects (e.g., If the pedestrian had not crossed the road, the accident would have been avoided). The fact that these drivers, in conditions of low control, demonstrate significant relationships between a higher number of errors committed and a higher level of shame leads us to suggest that it is shame, with the non-acceptance of internal limitations or the ability of the driver, which might explain that these drivers evade their responsibilities and indicate that they could have obtained better results if there had been external factors involved (i.e., upward counterfactuals). Fee and Tangney (2000) indicated that shame proneness was related to procrastination tendencies, whereas guilt proneness was not. These authors indicated that procrastination was a means of self-protection.

**FIGURE 1:**
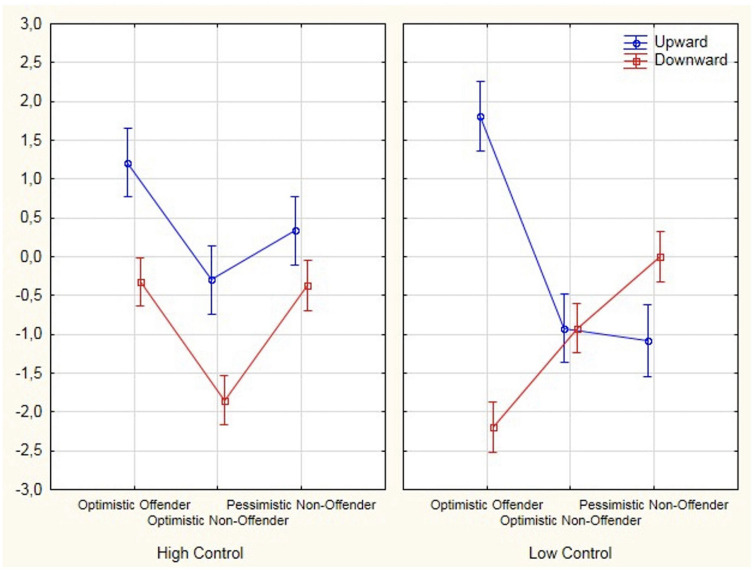
Effect of interaction between (upward and downward) counterfactual factor on study group and task condition.

Optimistic non-offender drivers recorded a different profile than the group previously commented. We have found that downward counterfactual thoughts require special attention because they are the ones that recorded differences depending on the control conditions (as shown in Figure 1). McMullen and Markman (2000) have commented that if a downward counterfactual conjures a negative affect by considering the real possibility that the outcome could have been far worse, it could serve as an admonition to change the behavior of one. We have observed that in situations of higher control when these drivers thought things could have been worse had they not taken different measures (i.e., downward counterfactual), they also experienced more guilt than shame. Following the theories of counterfactual reasoning posited by Mandel (2005), subjects may focus not only on how the events could have been avoided but also on how they were caused. The fact that these subjects report greater guilt than shame may explain the regret they may experience when, despite controlling the situation, they perceive their failures and responsibility (Tangney and Dearing, 2003; Mandel and Dhami, 2005).

Under conditions of low control, we can see how drivers recorded higher levels of shame and guilt, experienced simultaneously (as shown in Figure 1). Similar to the study of Tangney et al. (2007), we contend that guilt becomes maladaptive when merged with shame. Alicke et al. (2008) have reported the assertion among counterfactual reasoning theorists that the emotional response of an observer to an event is amplified when accompanied by the knowledge that an outcome could have been averted. Under situations of low control, in which bad results are also obtained, it is not functional to think that the results would have been worse if the driver had not carried out actions to avoid negative results (i.e., downward counterfactuals), and these could have been avoided. Furthermore, under this condition, downward counterfactual thoughts did not show the functionality indicated by Sanna (1998), Sanna et al. (2001). They gave this thought a restorative mood function, which could also be used to feel better, or as a feeling of relief.

**FIGURE 2:**
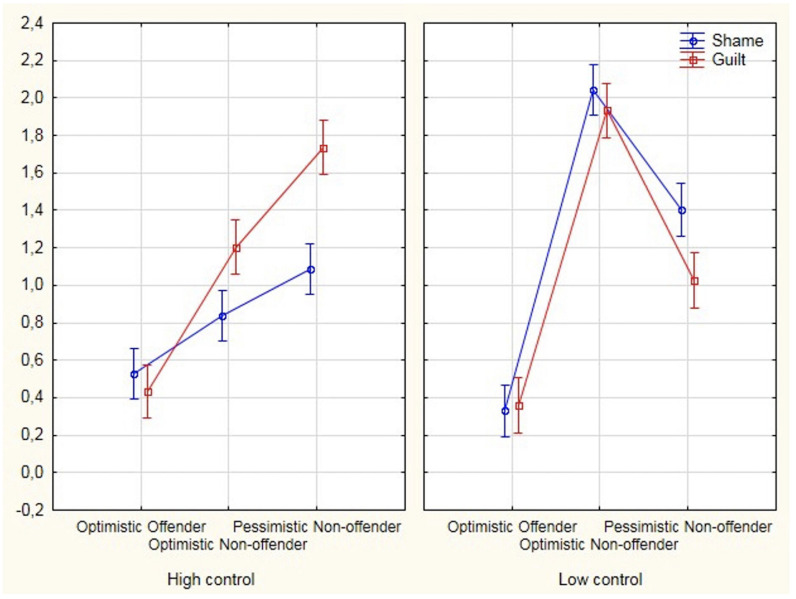
Effect of interaction between (guilt and shame) negative emotion factor on study group and task condition.

In the group of pessimistic non-offenders, upward counterfactual thoughts varied, depending on the conditions (as shown in Figure 1). When these drivers generated this type of thoughts, they attributed the result to their abilities under conditions of low controllability (i.e., an internal, stable, and uncontrollable factor). Under this condition, they tended to feel shame. Similar to the study of Tracy and Robins (2007), we understand that shame appears when the responsibility for the result is focused on the ability of a person, not on a specific behavior “If I were a more skilled driver, I would not have had this crash.” This alternative clearly emphasizes an internal factor, which underpins a negative evaluation of the self-image of the driver. This emotion may evoke more reticent behavior, which serves to distance the driver from the situation (Tangney and Dearing, 2003). Various authors (Sanna, 1998; Nasco and Marsh, 1999; Branscombe et al., 2003) have reported that the preparative function of the upward counterfactuals is observed among individuals whose self-efficacy is high, but not low. Markman and Miller (2006) have found that people with moderate depression did not obtain benefits when considering how they could have prevented the negative results. This leads us to suggest that upward counterfactuals can become dysfunctional since they would foster the emotion of shame, as shown in Figure 2.

As we have been commenting, the functionality of counterfactual thoughts and negative emotions appears under situations with unfavorable outcomes, which elicit a more causal than the favorable type of reasoning (Boninger et al., 1994). The negative emotions in optimistic offenders do not vary under the different control conditions. This is not the case in the other two groups. Similar to the study of Murrar et al. (2019), we believe that shame can negatively influence behavior, while guilt can do so positively. The drivers show the influence of shame under the condition of low control on both optimistic and pessimistic non-offenders. In turn, the role of guilt appears in the high-control condition of these groups. The level of guilt is heightened when people imagine how a change in their actions could have produced a different result; for example, “I would not have been fined if I had not speeded.” In contrast, the level of shame is heightened when they imagine how a change in their personality could have produced a different result; for example, “I would not have had the accident if I were not so clumsy” (Niedenthal et al., 1994). As it is understandably difficult to change personality traits, shame is, therefore, described as a maladaptive emotion since it promotes withdrawal and hinders the reparation process, while guilt is seen as an adaptive emotion, as it motivates repair (Tangney et al., 2014; Carpenter et al., 2016).

### Limitations and Future Directions

Few studies have focused on analyzing counterfactual thoughts and their link to negative emotions among optimistic offenders compared with non-offender drivers. Nevertheless, certain limitations must be taken into account when interpreting these results; the first of which is related to the groups of drivers. The scientific literature follows a theoretical continuum between optimism and pessimism, featuring additional categories, which may also be differentiated regarding the degree of perceived personal control over a positive outcome. There are, on the one hand, unrealistic optimistic drivers who consider themselves to possess extreme internal controllability and to be exempt from external difficulties that might influence the achievement of their objectives. There is also, however, a “defensive pessimistic” category that can be found halfway between the two extremes of optimism and pessimism. These individuals attempt to cope with their anxiety by anticipating the negative aspects of a situation, while simultaneously planning measures that enable them to achieve the desired outcome (Fernández and Bermúdez, 2001; del Valle and Mateos, 2008; del Valle, 2019). The second limitation involved ignoring pessimistic dispositional offenders because very few drivers view that category according to expectations. We acknowledge that data for this group would provide important information in our effort to interpret the results obtained in this study on counterfactual thinking and the negative emotions of pessimistic drivers. The third and final limitation is related to the use of objective measures to evaluate emotions linked to counterfactual thoughts. This article has focused on the evaluation of negative emotions. The experimental model in future studies should incorporate measures of positive emotions. We believe that positive emotions (e.g., satisfaction) could be characteristic of the optimistic group of offenders.

The importance of this study is in establishing the cognitive profile of drivers whose behavior behind the wheel is considered by the Directorate General for Traffic (DGT) in Spain to be unacceptable and in need of change. Our study focused on a group of optimistic offender drivers and on analyzing the role of causal attributions in counterfactual thinking as well as the emotions caused by conditions of induced control. The study of thoughts of this nature focuses on the subjective perception of control that drivers think they have. In turn, it could explain why individuals drive dangerously in a more or less voluntary manner. Awareness of how this cognitive process works and its impact on driving could foster a change in dangerous driving habits.

#### Implications

Our findings should help to enhance the effectiveness of driver reeducation courses, following a loss of license points, and reduce the likelihood of a relapse, which could advance efforts to prevent road accidents. We maintain that these courses are highly non-specific and do not address the characteristics of drivers that have lost all their license points. Therefore, identifying the cognitive and emotional profiles of drivers and seeing how they are connected to driving behavior is an important first step in an endeavor to develop strategies and reduce dangerous driving.

In conclusion, it is a fact that highly skilled drivers, or those who believe they are, may, be at greater risk as a result of their propensity to take risks on the road. Overestimating their abilities and not understanding their limitations are critical safety factors (Gregersen, 1996), especially when these drivers tend not to experience negative emotions when they fail. This is the case of optimistic offender drivers, unable to determine the causes of their failures. Regardless of the conditions, these drivers attribute their errors to external and uncontrollable factors, which means they do not experience, as far as possible, negative emotions in the face of failure. These failures will, therefore, tend to be repeated, and the planning of future actions will not be effective (Petrocelli and Harris, 2011), which entails a loss of opportunities to make future improvements, as indicated by different authors (Allison et al., 1996; Markman et al., 2007).

## Data Availability Statement

The raw data supporting the conclusions of this article will be made available by the authors, without undue reservation.

## Ethics Statement

The studies involving human participants were reviewed and approved by University of Salamanca. The patients/participants provided their written informed consent to participate in this study.

## Author Contributions

The author confirms being the sole contributor of this work and has approved it for publication.

## Conflict of Interest

The author declares that the research was conducted in the absence of any commercial or financial relationships that could be construed as a potential conflict of interest.

## Publisher’s Note

All claims expressed in this article are solely those of the authors and do not necessarily represent those of their affiliated organizations, or those of the publisher, the editors and the reviewers. Any product that may be evaluated in this article, or claim that may be made by its manufacturer, is not guaranteed or endorsed by the publisher.
