# Supplementary material for: Guilt and Shame of What Might Have Been in Optimistic Offender Drivers
Source: Front Psychol. 2021 Oct 7;12:668138. doi: 10.3389/fpsyg.2021.668138 (PMC8529183; doi:10.3389/fpsyg.2021.668138)
Supplement: Supplementary file 1 [file Table_1.docx]

Coeficientes de correlación lineal de Pearson entre Study Group and Task Condition for *the following variables: Mistake, Guilt, and Shame*

| Study Group | Task Condition |  |  | Variables |  |
| --- | --- | --- | --- | --- | --- |
|  |  |  | Mistake | Guilt | Shame |
| Optimistic Offender | High control  (n = 28) | Mistake |  |  |  |
|  |  | Guilt | -.180 |  |  |
|  |  | Shame | -.195 | -.124 |  |
|  | Low control  (n = 26) | Mistake |  |  |  |
|  |  | Guilt | .318 |  |  |
|  |  | Shame | .515** | .259 |  |
| Optimistic Non-Offender | High control  (n = 28) | Mistake |  |  |  |
|  |  | Guilt | -.119 |  |  |
|  |  | Shame | -.150 | -.216 |  |
|  | Low control  (n = 27) | Mistake |  |  |  |
|  |  | Guilt | .212 |  |  |
|  |  | Shame | -.095 | .148 |  |
| Pessimistic Non-Offender | High control  (n = 27) | Mistake |  |  |  |
|  |  | Guilt | -.023 |  |  |
|  |  | Shame | .103 | -.306 |  |
|  | Low control  (n = 25) | Mistake |  |  |  |
|  |  | Guilt | .113 |  |  |
|  |  | Shame | .180 | .006 |  |

*Note. *p< .05, **p< .001,*
